# The Role of Financial Difficulties as a Mediator between Physical Symptoms and Depression in Advanced Cancer Patients

**DOI:** 10.3390/curroncol30060429

**Published:** 2023-06-12

**Authors:** Eun Mi Lee, Paula Jiménez-Fonseca, Raquel Hernández, Patricia Cruz-Castellanos, Ana Fernández-Montes, Jacobo Rogado, Mireia Gil-Raga, Mónica Antoñanzas, Helena López-Ceballos, Caterina Calderon

**Affiliations:** 1 Faculty of Psychology, University of Barcelona, 08007 Barcelona, Spain; 2 Department of Medical Oncology, Hospital Universitario Central de Asturias, Instituto de Investigación del Principado de Asturias, ISPA, 33011 Oviedo, Spain; 3 Department of Medical Oncology, Hospital Universitario de Canarias, 38320 Tenerife, Spain; 4 Department of Medical Oncology, Hospital General Universitario de Ciudad Real, 13005 Madrid, Spain; 5 Department of Medical Oncology, Complejo Hospitalario Universitario de Ourense, 32005 Ourense, Spain; 6 Department of Medical Oncology, Hospital Infanta Leonor, 28031 Madrid, Spain; 7 Department of Medical Oncology, Consorci Hospital General Universitario de Valencia, 46014 Valencia, Spain; 8 Department of Medical Oncology, Hospital Clínico San Carlos, 28040 Madrid, Spain; 9 Department of Medical Oncology, Hospital General Virgen de la Luz, 16002 Cuenca, Spain

**Keywords:** financial difficulties, symptoms, depression, cancer, quality of life

## Abstract

Financial difficulties experienced by cancer patients negatively impact the mental health of the patients. The objective of this study was to examine the mediating role of financial difficulties between physical symptoms and depression in patients with advanced cancer. A prospective, cross-sectional design was adopted in the study. The data were collected from 861 participants with advanced cancer in 15 different tertiary hospitals in Spain. The participants’ socio-demographic characteristics were collected using a standardized self-report form. Hierarchical linear regression models were used to explore the mediating role of financial difficulties. In the results, 24% of patients reported a high level of financial difficulties. Physical symptoms were positively associated with financial difficulties and depression (β = 0.46 and β = 0.43, respectively), and financial difficulties was positively associated with depression (β = 0.26). Additionally, financial difficulties played a role in explaining the relationship between physical symptoms and depression, showing a standardized regression coefficient of 0.43 which decreased to 0.39 after the financial difficulties were controlled. Healthcare professionals should consider the importance of providing financial resources and emotional support to help patients and their families cope with the financial burden associated with cancer treatment and its symptoms.

## 1. Introduction

Cancer represents the most expensive disease in terms of average expenditure per patient [1]. Individuals with advanced cancer undergoing treatment often experience the consequences of high costs, such as medications not covered by health insurance, the need of in-home assistance for daily activities, out-of-pocket expenses, loss of income due to inability to work during treatment, and other expenses such as acquiring a new specific diet, transportation, or lodging if treatment is performed away from home [1,2,3]. The term “financial difficulties” have been coined to describe both the objective financial burdens of healthcare as well as the subjective distress experienced by patients [1].

Financial difficulties can generate significant stress, anxiety, and depression in individuals and their families, thereby affecting their quality of life and exacerbating both the physical and emotional symptoms of the patients [4,5]. Additionally, financial constraints can limit access to medical resources, including medications and support services, which can cause a negative impact on treatment adherence, symptom burden, and patient prognosis [2,3,6]. Financial burdens can exceed 20% of income [7], potentially increasing the mortality rate of the patient [8].

Several studies have shown that there are multiple risk factors associated with financial difficulties in cancer patients [3]. Among them, predominant demographic factors have been identified, such as female sex, younger age, current employment, socioeconomic status, insurance coverage, and distance from the treatment center [2,9]. For example, women over 55 years old with breast cancer compared to women under 55 years old usually experience greater financial difficulties, disruptions in their daily lives, and lower levels of emotional well-being [10]. Moreover, it has been shown that younger and unmarried patients are more likely to report financial difficulties [11].

The interactions between financial difficulties, physical symptoms, and depression are complex. Although several studies have found an association between higher financial burden, depression, and a reduction in health-related quality of life, particularly in emotional well-being [2,4], little is known about how economic difficulties, physical symptoms, and depression are related, especially in patients with advanced cancer. Therefore, research is needed to evaluate how these factors may interact with each other. The main objective of this research was to examine the mediating role of financial difficulties between physical symptoms and depression in patients with advanced cancer. It is plausible to assume that patients with a greater burden of symptoms (such as fatigue, pain, or appetite loss) may be forced to reduce their working hours or be unable to return to work, which could exacerbate the economic pressure and contribute to an increase in depression.

## 2. Materials and Methods

### 2.1. Study Design and Population

The study was a prospective, cross-sectional investigation, carried out across 15 medical oncology departments at various hospitals in Spain from February 2020 to October 2021 (Table A1, see Appendix A section). Our aim was to include the oncology departments from various regions of Spain for a representative sample. We selected departments with a high inflow for advanced cancer patients and adequate clinical and necessary resources. Once an oncology department head expressed interest, they received detailed research objective explanations. The study was approved by the Ethics Review Committee of each participating institution and by the Spanish Agency of Medicines and Health Products (AEMPS; identification code: ES14042015). Spain has approximately 210 medical oncology departments distributed across its 17 autonomous communities. The study included approximately 7.1% of hospitals from 9 autonomous communities. Patients were recruited during their first visit to the medical oncologist where the diagnosis, disease stage, and available systemic antineoplastic treatments were determined. To be eligible for the study, the participants had to be 18 years or older, with a histological confirmation of advanced cancer, and could not be a candidate for surgery or other curative therapies. Patients were excluded if their physical condition, age, or comorbidity contraindicated antineoplastic treatment as per the attending oncologist’s opinion. Participants who had received cancer treatment for another advanced cancer within the past two years, or who had underlying medical, sociological, family, or personal conditions that could hinder their participation in the study were also excluded. Furthermore, individuals with cognitive impairment or severe deterioration of their general status, as well as those who were unable to understand or reason through the questionnaires were excluded. All participants provided informed consent before enrollment. The data collection involved completing questionnaires and collecting clinical information from interviews and medical records. The data collection process was similar in all participating hospitals and the patient data were obtained from the institutions where they received treatment. The participation was voluntary and anonymous, which did not affect the patients’ care. The medical oncologists updated and collected data using a web-based platform (www.neoetic.es accessed on 2 May 2023). Out of 918 screened patients, 861 were eligible for analysis, while 57 were excluded due to failure to meet the inclusion criteria (*n* = 10) or exclusion criteria (*n* = 9) or had incomplete data (*n* = 38) at the time of analysis.

### 2.2. Description of Variables

The extent to which cancer was impacted by financial difficulties was determined based on the EORTC-QLQ-C30 questionnaire, which measures perceived financial difficulties [12]. Within the questionnaire, there was a specific item that assessed the effect of cancer on patients’ financial difficulties: ‘Has your physical condition or medical treatment caused you financial difficulties?’ Based on the responses obtained, patients were divided into two groups: Not at All/Little (FD0) and Quite a Bit/Very Much (FD1). The raw score, as a continuous variable, was linearly transformed into a scale of 0–100, as suggested by the authors of [12]. In the study, the cut-off used was similar to that used by other authors [13], and the Cronbach’s alpha for the scale was 0.88.

The physical symptoms was measured using the subscale “physical symptoms” of the EORTC Quality of Life Questionnaire C30 (QLQ-C30) version 2 [12]. The EORTC physical symptoms subscale is useful for measuring the symptom burden experienced by cancer patients, which also evaluates the effectiveness of treatments in relieving symptoms. These physical symptoms include fatigue, pain, nausea/vomiting, appetite loss, constipation, diarrhea, sleep problems, and dyspnea. The physical symptoms subscale consists of 13 items that patients rate on a 4-point scale, which ranges from “not present” to “very intense”. The total score of the subscale is calculated as the average of the scores of all the items included in the subscale. All scores were linearly converted on a scale from 0 to 100 [12], in which a higher score indicates a greater presence and intensity of symptoms. In the Spanish sample, the reliability of the scale was 0.86 [14].

The assessment of depression was conducted using the Brief Symptom Inventory (BSI), a 6-item depression subscale [15], which specifically evaluates physical symptoms of disaffection and dysphoric mood, such as self-deprecation, anhedonia, hopelessness, and suicidal ideation. Raw scores were then converted to T-scores [15]. In Spanish cancer patients, the internal consistency of the scale was found to be 0.75 [16].

### 2.3. Statistical Methods

To analyze the demographic and clinical characteristics of the patients, the calculation of descriptive statistics and frequency distributions was conducted. Chi-square analyses were conducted to compare the differences in demographic, clinical, and psychological features between low and high scores of financial difficulties in patients. To determine whether financial difficulties mediated the association between physical symptoms and depression in advanced cancer patients, mediation analyses were carried out. Pearson’s correlation was used to examine the correlations between continuous variables. Hierarchical regression analysis was used to explore the mediating effects of financial difficulties on the relationship between physical symptoms and depression. According to Baron and Kenny’s mediation technique [17], the following conditions should be met: (1) the independent variable (physical symptoms) is significantly related to the dependent variable (depression); (2) the independent variable (physical symptoms) is significantly related to the mediator (financial difficulties); (3) the mediator (financial difficulties) is significantly related to the dependent variable (depression), considering the effect of the independent variable (physical symptoms) on the dependent variable (depression) when including the mediator (financial difficulties) to the model. Moreover, Sobel’s test was performed to estimate the mediation effect. The IBM-SPSS 29.0 statistical software package by IBM Corp. in Armonk, NY, USA including IBM SPSS Statistics for Windows, was utilized for the analyses.

## 3. Results

### 3.1. Sociodemographic and Clinical Characteristics

A total of 861 patients were enrolled in the study, with 54% being male and 46% female. More than half of the participants were aged 65 years or older (57%), and the majority were married or in a partnership (69%). The most prevalent tumor sites were bronchopulmonary (30%), colorectal (17%), and pancreatic (10%), with 80% of patients having metastatic disease at the time of diagnosis. The most frequent treatment received was chemotherapy (54%), followed by immunotherapy (7%) and targeted therapies (6%). At the time of diagnosis, 62% of participants had an Eastern Cooperative Oncology Group (ECOG) status greater than 1, and more than half of the patients (55%) had an estimated survival of 18 months or more.

### 3.2. Financial Difficulties and Clinical–Demographic Characteristics

Out of all the participants, 76% (*n* = 653) reported low levels of financial difficulties, while 24% (*n* = 208) reported high levels of financial difficulties. When examining the association between financial difficulties and clinical–demographic characteristics, women (*X*^2^ = 10.031, *p* = 0.002) aged 65 years or older (*X*^2^ = 54.432, *p* = 0.001), and women with a high school education or a higher education level (*X*^2^ = 625.624, *p* = 0.001) reported lower levels of financial difficulties. However, there was no significant relationship between financial difficulties and marital status, tumor site, histology, stage, treatment modality, baseline ECOG status, or estimated survival (Table 1).

### 3.3. The Mediating Role of Financial Difficulties in the Relationship between Physical Symptoms and Depression

Table 2 shows the mean, standard deviations, and Pearson correlation coefficients of the variables. All variables were treated as continuous variables. The mean scores for financial difficulties (as a continuous variable), physical symptoms, and depression were 19.2 ± 32.6, 49.5 ± 21.4, and 63.2 ± 7.1, respectively. Significant positive correlations were found among all psychological variables in the expected direction. Financial difficulties were positively correlated with physical symptoms and depression, and physical symptoms was positively correlated with depression, meeting the first two conditions of Baron and Kenny’s technique.

**Figure 1:**
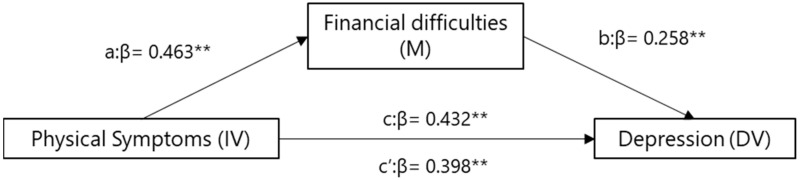
Mediation model for financial difficulties, symptoms, and depression. a = direct effect of independent variable (IV) on mediator (M). b = direct effect of mediator on dependent variable (DV). c = direct effect of IV on DV. c′ = indirect effect of IV on DV. ** *p* < 0.01.

Hierarchical linear regression analyses were conducted to investigate the mediating role of financial difficulties, as shown in Figure 1. The results indicated that physical symptoms were positively associated with financial difficulties and depression (β = 0.46, *p* = 0.001; β = 0.43, *p* = 0.001, respectively), and financial difficulties was positively associated with depression (β = 0.26, *p* = 0.001). Additionally, financial difficulties partially mediated the association between physical symptoms and depression, with its standardized regression coefficient (β) decreasing from 0.43 to 0.39 (Sobel test, *z* = 2.26, *p* = 0.02).

## 4. Discussion

To date, this study is the first to investigate financial difficulties in individuals with advanced cancer and to examine the mediating role of financial difficulties between physical symptoms and depression in a Spanish population. According to the findings, 24% of patients with advanced cancer reported having experienced financial difficulties. There were positive correlations between financial difficulties, physical symptoms, and depression. As anticipated, financial difficulties played a mediating role in the relationship between physical symptoms and depression.

The fact that 24% of cancer patients reported having experienced financial difficulties suggests that this is a common and significant issue among those who suffer from this disease. Furthermore, the presence of positive correlations between financial difficulties, physical symptoms, and depression indicates that these factors may be interrelated and negatively impact the mental health of patients. In recent years, numerous psychosocial studies have been conducted on cancer patients [2,3,5]. Financial difficulties and physical symptoms have been identified as risk factors for depression [13,18,19,20]. In the current study, the results suggest that financial difficulties are an important factor in the relationship between physical symptoms and depression. In particular, financial difficulties may mediate the association between physical symptoms and depression, which implies that addressing financial difficulties could be an effective way to reduce levels of depression in patients with advanced cancer [6,9]. A systematic review of studies on advanced cancer detected that anxiety is related to financial difficulties, such as struggling to make ends meet, struggling to pay bills, or having limited access to financial resources [5,11]. In the context of cancer, financial difficulties may also arise from the costs of medical treatments, such as high co-pays or out-of-pocket expenses, and from loss of income due to inability to work during treatment [2,3]. Financial difficulties can generate significant stress, anxiety, and depression in individuals and their families [4,6].

The current study’s results indicate that female individuals over 65 years old, and those with a high school education or higher education level reported higher levels of financial difficulties. These findings are consistent with previous research that suggests that women and seniors are more vulnerable to financial hardships, which can negatively affect their mental health and quality of life [21,22,23]. Financial difficulties can cause stress and anxiety in patients with advanced cancer, leading to a deterioration in their physical and mental well-being [4,6,24]. In addition, financial constraints can limit access to critical healthcare resources, such as medication, consultations, and support services, which can compromise treatment outcomes and prognosis [4,8]. Consequently, patients may be reluctant to seek medical attention or comply with prescribed treatments due to financial constraints [9].

The current study has both strengths and limitations. One limitation is that the design of the study was cross-sectional, which precludes drawing causal inferences between the studied variables. In future research, longitudinal cohort studies will be needed to confirm the current findings. Another limitation is that all data were obtained through self-reporting questionnaires, which may introduce response biases. The participants may have overestimated or underestimated the relationship between variables. Additionally, the study did not examine differences in behavior between patients with different types of neoplasms, and therefore, the influence of tumor type was not analyzed in the findings. It would be interesting to conduct this analysis in patients with specific types of cancer. Finally, while the sample was representative for the Spanish population, caution should be exercised when generalizing the results to other cultures and societies.

## 5. Conclusions

In conclusion, the clinical implications of financial difficulties in patients with advanced cancer are significant. Patients who experience financial difficulties may have increased stress and depression, which can affect their quality of life and worsen their physical and emotional symptoms. Moreover, financial difficulties can limit access to critical medical resources, including medications and support services, which could negatively impact the quality of care and prognosis for patients.

Consequently, it is important for healthcare professionals to recognize the potential impact of financial difficulties on patients with advanced cancer and provide adequate support and resources to address their financial concerns. This may include financial counseling, assistance programs, and advocacy for improved access and affordable healthcare resources. It may also be helpful for healthcare professionals to discuss the possibility of financial difficulties with patients before it becomes a significant concern for the patient and their family.

## Figures and Tables

**Table 1:** Differences in demographic and clinical characteristics among financial difficulties (*n* = 861).

Variables	Total Sample *n* (%) 861 (100%)	Low Financial Diff. *n* (%) 653 (76%)	High Financial Diff. *n* (%) 208 (24%)	*X* ^2^	*p* Value	Standardized % by Sex and Age
Sex						
Male	467 (54)	374 (57)	93 (45)	10.031	0.002	54%
Female	394 (46)	279 (43)	115 (55)			46%
Age						
<65 y	369 (43)	234 (36)	135 (65)	54.432	0.001	43%
≥65 y	492 (57)	419 (64)	73 (35)			57%
Marital status						
Married or part.	592 (69)	455 (70)	137 (66)	1.068	0.301	-
No partner	269 (31)	198 (30)	71 (34)			-
Educational level						
Primary	442 (51)	367 (56)	75 (36)	25.624	0.001	-
≥High school	419 (49)	286 (44)	133 (64)			-
Tumor site						
Bronchopulmonary	257 (30)	198 (30)	59 (28)	1.923	0.586	-
Colorectal	143 (17)	108 (17)	35 (17)			-
Pancreas	83 (10)	67 (10)	16 (8)			-
Others	378 (44)	280 (43)	98 (47)			
Histology						
Adenocarcinoma	553 (64)	420 (64)	133 (64)	0.010	0.921	-
Others	308 (36)	233 (36)	75 (36)			-
Stage						
Locally Advanc.	173 (20)	128 (20)	45 (22)	0.406	0.524	-
Metastatic (IV)	688 (80)	525 (80)	163 (78)			-
Type of treatment						
Chemotherapy	464 (54)	358 (55)	106 (51)	5.927	0.115	-
Immunotherapy	59 (7)	48 (7)	11 (5)			-
Targeted therapy	52 (6)	33 (5)	19 (9)			-
Others	286 (33)	214 (33)	72 (34)			-
ECOG performance status						
0	326 (38)	247 (38)	79 (38)	0.002	0.968	-
1 or more	535 (62)	406 (62)	129 (62)			-
Estimated Survival						
<18 months	391 (45)	309 (47)	82 (39)	3.869	0.056	-
≥18 months	470 (55)	344 (53)	126 (61)			-

**Table 2:** Correlations between financial difficulties, physical symptoms, and depression.

	Mean	SD	Financial Diff.	Physical Symptoms	Depression
Financial diff.	19.2	32.6	1		
Physical Symptoms	49.5	21.4	0.463 **	1	
Depression	63.2	7.1	0.258 **	0.432 **	1

** *p* < 0.01.

## Data Availability

The datasets generated during and analyzed during the current study are not publicly available for reasons of privacy. They are, however, available (fully anonymized) from the corresponding author on reasonable request.

## Appendix A

**Table A1:** List of 15 centers that participated in the study.

(1) Hospital Universitario de Canarias, Tenerife
(2) Hospital Universitario La Paz, Madrid
(3) Hospital Universitario Central de Asturias, Oviedo
(4) Complejo Hospitalario Universitario de Ourense
(5) Hospital Universitario Infanta Leonor, Madrid
(6) Consorcio Hospital General Universitario de Valencia, Valencia
(7) Hospital General Virgen de la Luz, Cuenca
(8) Hospital Provincial de Castellón, Castellón
(9) Hospital General Universitario de Elche, Elche
(10) Hospital Universitario Clínico San Carlos, Madrid
(11) Hospital San Pedro de Alcántara, Cáceres
(12) Hospital Universitario Morales Meseguer, Murcia
(13) Hospital Quironsalud Sagrado Corazón de Sevilla, Sevilla
(14) Hospital General Universitario Santa Lucia, Cartagena
(15) Hospital General de Ciudad Real, Ciudad Real
